# Coherence between Rat Sensorimotor System and Hippocampus Is Enhanced during Tactile Discrimination

**DOI:** 10.1371/journal.pbio.1002384

**Published:** 2016-02-18

**Authors:** Natalia Grion, Athena Akrami, Yangfang Zuo, Federico Stella, Mathew E. Diamond

**Affiliations:** Tactile Perception and Learning Lab, International School for Advanced Studies (SISSA), Trieste, Italy; Brain Mind Institute, SWITZERLAND

## Abstract

Rhythms with time scales of multiple cycles per second permeate the mammalian brain, yet neuroscientists are not certain of their functional roles. One leading idea is that coherent oscillation between two brain regions facilitates the exchange of information between them. In rats, the hippocampus and the vibrissal sensorimotor system both are characterized by rhythmic oscillation in the theta range, 5–12 Hz. Previous work has been divided as to whether the two rhythms are independent or coherent. To resolve this question, we acquired three measures from rats—whisker motion, hippocampal local field potential (LFP), and barrel cortex unit firing—during a whisker-mediated texture discrimination task and during control conditions (not engaged in a whisker-mediated memory task). Compared to control conditions, the theta band of hippocampal LFP showed a marked increase in power as the rats approached and then palpated the texture. Phase synchronization between whisking and hippocampal LFP increased by almost 50% during approach and texture palpation. In addition, a greater proportion of barrel cortex neurons showed firing that was phase-locked to hippocampal theta while rats were engaged in the discrimination task. Consistent with a behavioral consequence of phase synchronization, the rats identified the texture more rapidly and with lower error likelihood on trials in which there was an increase in theta-whisking coherence at the moment of texture palpation. These results suggest that coherence between the whisking rhythm, barrel cortex firing, and hippocampal LFP is augmented selectively during epochs in which the rat collects sensory information and that such coherence enhances the efficiency of integration of stimulus information into memory and decision-making centers.

## Abstract 2

In rats, the rhythms of whisking and hippocampal theta become coherent precisely when rats approach and explore a texture; higher coherence enhances the identification of texture.

## Author Summary

Many regions of the mammalian brain exhibit oscillations in electrical activity. In rats, the 5–12 Hz theta rhythm is present in the hippocampus and in diverse areas of the cerebral cortex. What is the function of this rhythm? One proposal is that the exchange of information between two brain regions is facilitated whenever their respective oscillations are coherent. To test this idea, we ask whether theta oscillation in the hippocampus, a crucial memory structure located deep in the brain, is coherent with the rat’s rhythm of moving its whiskers and sensing the physical environment with them. We acquired hippocampal local field potentials (LFP)—extracellular voltage fluctuations within a small volume—while rats classified textures using cyclical whisker motion (“whisking”). At the moment of texture palpation, coherence between whisking and hippocampal theta oscillations increased by nearly 50%. At the same time, neuronal firing in sensory cortex became more phase-locked to the hippocampal theta oscillations. Rats identified the texture more rapidly and with lower error likelihood on trials characterized by an increase in hippocampal theta-whisking coherence during texture palpation. These results suggest that, as rats collect touch signals, enhanced coherence between the whisking rhythm, sensory cortex, and hippocampal LFP facilitates the integration of sensory information into memory and decision-making centers in the brain.

## Introduction

Fluctuations in local field potential (LFP) affect a neuronal population’s likelihood of spike output and its sensitivity to synaptic inputs [1–3]). When the oscillations of two brain regions are coherent, the respective neuronal populations might be functionally coupled to allow efficient transmission of signals [4]. Moments of high coherence could permit target neurons to fire at low energy cost [5]. The degree of LFP coherence between two populations depends on the animal’s state [6,7], and heightened coherence has been observed in various cognitive tasks [8–10].

Oscillations in the theta frequency range (5–12 Hz) are a prominent feature of hippocampal spiking and LFP as rats engage in a wide range of behaviors [11,12]. The effects of manipulating the theta rhythm suggest specific roles of different theta phases in encoding versus decoding processes [13]. In rats, the hippocampus and prefrontal cortex undergo intervals of coherent LFP oscillation during spatial navigation [14]. We hypothesize that not only prefrontal but also primary sensory regions of neocortex might be linked to hippocampus by coherent oscillations. The rat whisker sensorimotor system offers intriguing opportunities for testing this hypothesis because the system is entrained by a periodic rhythm, the forward-backward sweeping of the whiskers [15–17]. Though whisking and theta reside within the same 5–12 Hz frequency band, whisking does not depend on theta—ablation of the medial septum abolishes the theta rhythm while whisking remains intact [18,19]. A specific phase relationship between the hippocampal theta rhythm and whisking has been posited [20], yet coherence between sensorimotor system and hippocampus was not found to be higher than would be expected for independent oscillators when rats whisked in the air under conditions that involved no memory component [21]. These results, taken together, suggest independent rhythmic generators for whisking and the theta rhythm. Whether these generators become coherent, and if so, under what circumstances, remains to be elucidated.

Here we address this issue in the context of a whisker-mediated perceptual task. While rats carried out a texture discrimination task, we examined the relationships between three variables: (i) the LFP oscillation of the CA1 region of hippocampus, (ii) the cyclical forward and backward “whisking” motion, and (iii) the firing of neurons in barrel (primary somatosensory) cortex. Barrel cortex is the main gateway by which signals from the whiskers enter neocortex; from there, signals are relayed to the hippocampus [22] where they are integrated with spatial and other sorts of information [23]. We hypothesize that coherence between the whisking rhythm, barrel cortex firing, and hippocampal theta might be enhanced selectively during epochs in which the rat collects sensory information whose destination is the hippocampus.

## Results

### Overview of Experimental Design

**Fig 1:**
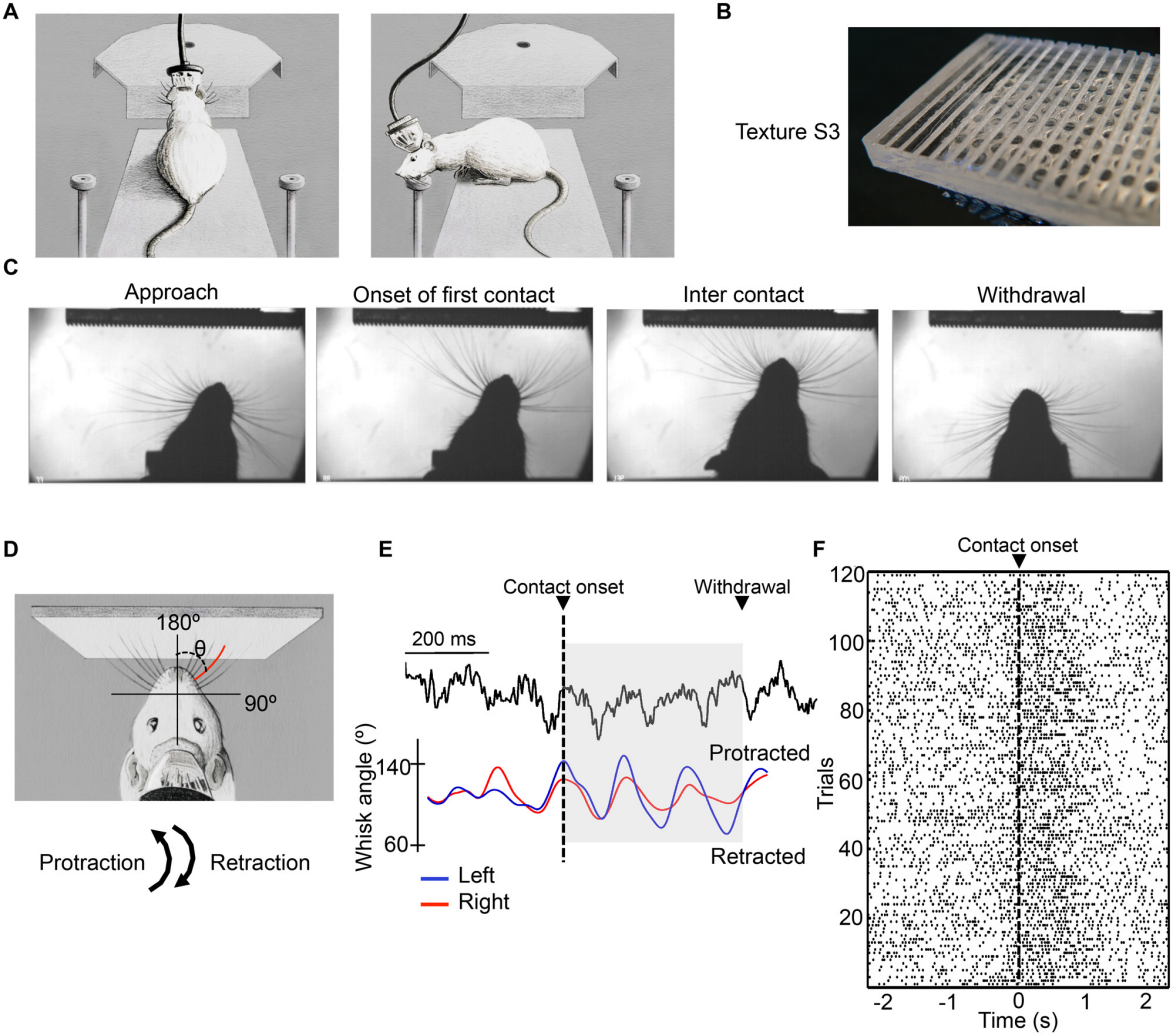
Experimental setup, behavior, and recordings. **(A)** Sketches of texture discrimination task. Left panel: the rat extends forward to identify the textured plate using its whiskers. Right panel: having identified the stimulus, the rat withdraws from the center and collects the reward at the left spout. **(B)** Photograph of texture S3, a Plexiglas plate with vertical grooves of 1 mm width and depth cut at intervals of 2 mm. **(C)** Sensorimotor behavior on a typical trial is characterized by four frames from high-speed video: approach (head, body, and whiskers all move forward), onset of first contact, inter contact (whiskers are retracted and detached from texture prior to the subsequent protraction and contact), and withdrawal (whiskers are retracted and rat’s head and body move backwards onto the platform). **(D)** Coordinate system to quantify whisking angle in the automatic whisker tracking program. **(E)** Simultaneous measurement of CA1 LFP and whisker mean angle extracted from a typical trial. Black line, LFP; blue line, left whiskers; red line, right whiskers. Whisking angle refers to coordinate system in **D**. **(F)** Raster plot of the spikes of a barrel cortex unit across trials from a single session. Trials are not labeled according to the stimulus. Spike trains are aligned to the onset of first contact, denoted 0 s. All data are available at http://figshare.com/s/99b31b8a567f11e5b81d06ec4bbcf141.

We trained seven rats to perform a tactile discrimination task (Fig 1A) in which they classified textured plates (Fig 1B, see Materials and Methods) touched by their whiskers. One of the plates, selected randomly, was presented on each trial, and the rat collected a water reward if it correctly turned to the side (left or right) associated with that plate (Table 1). Once rats performed correctly at least 65% of the time on three consecutive sessions, we implanted two arrays of tetrodes in order to measure LFP from dorsal hippocampus (CA1 region) and the spiking activity of barrel cortex neurons simultaneously. We also monitored whisking motion. On each trial, the rat’s actions were recorded (Fig 1C) and four behavioral episodes were extracted based on triggering of optic sensors and video analysis: “approach,” “touch,” “turn,” and “reward.” The mean value of touch episode length was 540 ms, and so 500 ms was selected as the analysis interval length for the episodes included in the analysis: “approach,” “touch,” and “reward” (see Materials and Methods).

**Table 1:** Side-texture associations of the tactile discrimination task.

Experiment	Textures associated with:	
	Reward to the right	Reward to the left
**Rat 1**	**S1**	**S2**
**Rat 2**	**S1**	**S2**
**Rat 3**	**S1**	**S3, S4**
**Rat 4**	**S1**	**S2**
**Rat 5**	**S3**	**S1**
**Rat 6**	**S3**	**S1**
**Rat 7**	**S3**	**S1**

**Fig 2:**
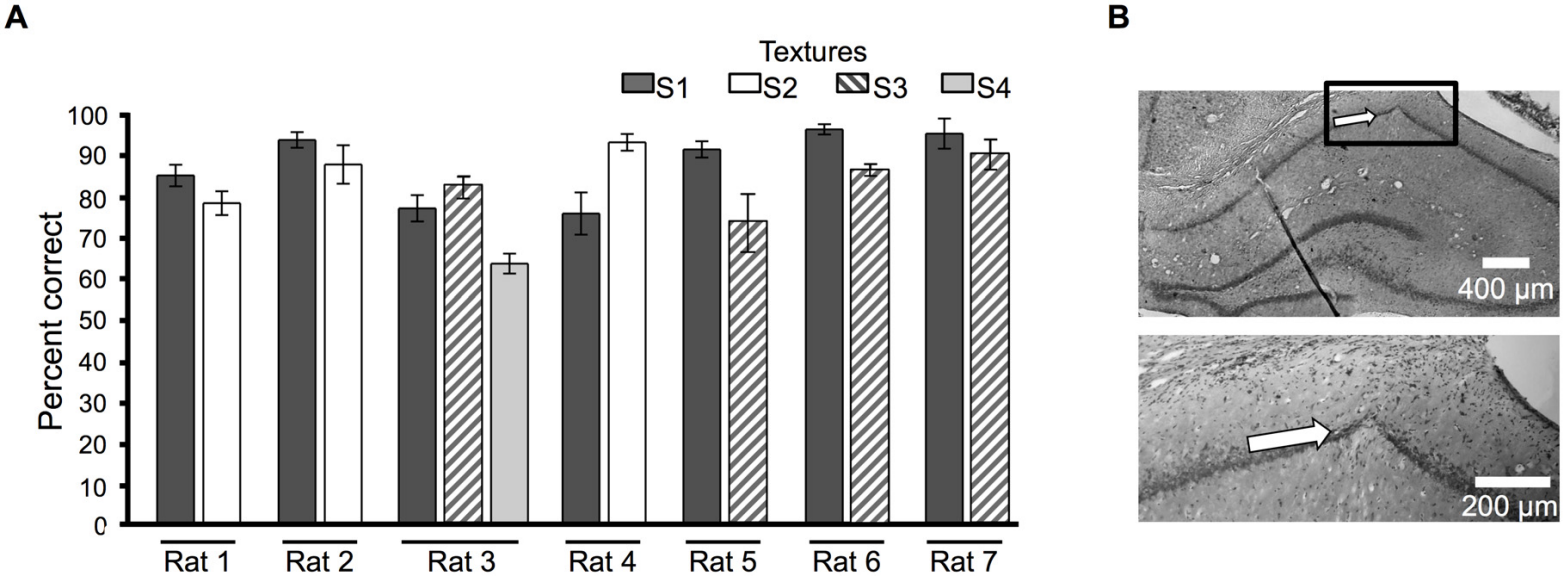
Behavioral performance and hippocampal histology. **(A)** Mean behavioral performance of each of seven tested rats during recording sessions. Values are mean percent correct trials ± standard error of the mean (SEM) across sessions, separated according to the stimulus presented. The number of sessions per rat included in the analysis was as follows: Rat 1: 5 sessions, 551 trials; Rat 2: 11 sessions, 1,020 trials; Rat 3: 2 sessions, 312 trials; Rat 4: 5 sessions, 541 trials; Rat 5: 5 sessions, 433 trials; Rat 6: 9 sessions, 1,127 trials; Rat 7: 4 sessions, 181 trials. **(B)** Histological section. White arrow indicates the electrode track within CA1 subfield of hippocampus. The boxed area of the upper photograph is shown at higher magnification below. All data are available at http://figshare.com/s/99b31b8a567f11e5b81d06ec4bbcf141.

We plotted the mean angle of the full set of whiskers [24]) across each trial (Fig 1D). This allowed us to compare the phase of whisking angle to the phase of hippocampal LFP (Fig 1E). Neurons in barrel cortex, recorded simultaneously, typically showed an increase in firing rate during texture exploration (Fig 1F). Performance during recording sessions was above 70% and better than chance (randomization test for every session of every rat, *p* < 10^−10^, see Materials and Methods for details) for all rats (Fig 2A). Tissue sections at the end of the experiment confirmed tetrodes position (Fig 2B).

Two of the animals (Rats 5, 6) were monitored outside the context of the texture discrimination task; they were placed on a square platform and foraged for cereal flakes. In these sessions, we recorded hippocampal LFP and whisking simultaneously and later classified each time period as an instance of walking or resting (see Materials and Methods).

### Hippocampal Theta Power during Texture Discrimination

**Fig 3:**
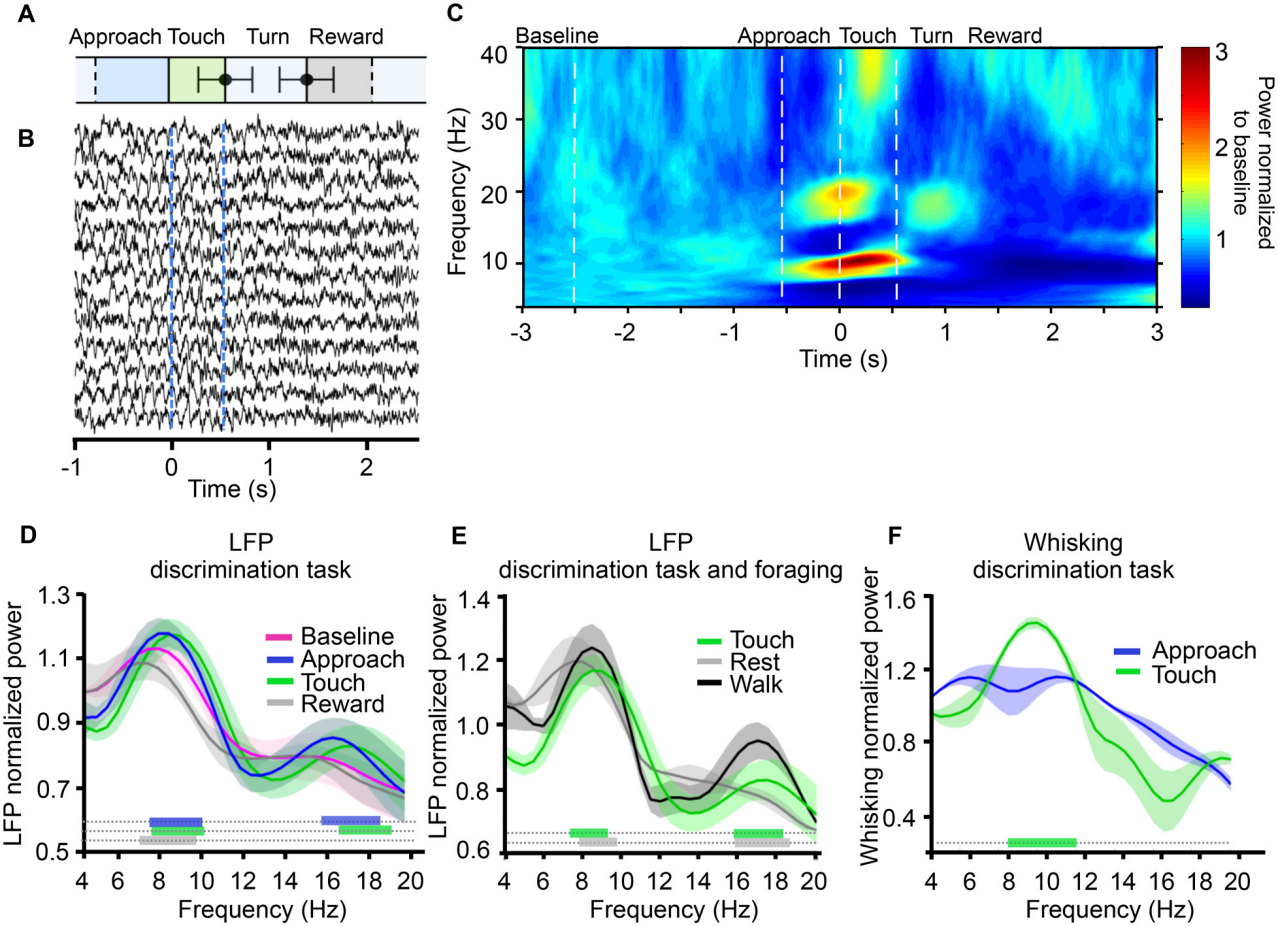
Hippocampal LFP during tactile discrimination task and foraging. **(A)** Time course of texture discrimination: bar lengths represent the average duration of each episode in the task, as labeled above. Touch (green bar) started at the onset of first contact and ended at the offset of final contact. Turn (light grey) started at the offset of final contact and ended at the arrival at the reward spout. Black points with error bars represent the mean ± standard deviation (SD) of ending time of touch (540 ± 224 ms) and turn (798 ± 395 ms) episodes across sessions and rats. Data were aligned to onset of first contact so SD = 0 at the boundary between approach and touch. Approach (blue) and reward (dark grey) had fixed duration but variable onset and offset, respectively (see Materials and Methods for analysis criterion), so their onset and offset are marked by dashed lines. **(B)** Hippocampal LFP of 13 trials (randomly chosen from one session) aligned to touch onset (0 s). **(C)** Averaged power spectrogram relative to baseline (defined as interval between -3 and -2.5 s) aligned to first contact (touch onset). Data are from same session as **B** but now all correct trials are included. Start and end of approach and touch are marked with dashed lines. **(D)** Grand average (*n* = 4 rats, also illustrated in **E** and **F**) of LFP power spectrum associated with different task episodes. In each animal LFP power was normalized to baseline power: LFP power value (10*log_10_(power)) from each task episode was divided by 4 Hz baseline LFP power (10*log_10_(4 Hz_baseline_)), and then averaged across animals. Shading is standard deviation (also in **E** and **F**). Color bars at the base of the figure indicate frequencies at which there was a significant power difference, versus baseline, during approach (blue), touch (green) and reward (grey) (randomization test, see Materials and Methods). **(E)** Grand average of LFP power spectrum during the discrimination task (touch episodes) and foraging (walk and rest episodes). Color bars indicate frequencies at which there was a significant power difference for the following comparisons: walk versus touch (green), and walk versus rest (grey). **(F)** Grand average of whisking power spectrum during the discrimination task (approach and touch episodes). Green bar indicates frequencies at which there was a significant power difference for approach versus touch, revealing the concentration at 8–12 Hz during touch. All data are available at http://figshare.com/s/99b31b8a567f11e5b81d06ec4bbcf141.

We first examined how the hippocampal LFP varied according to the animal’s ongoing behavior. Fig 3A shows key events of an exemplar trial, aligned to the time of first whisker contact with the stimulus. Error bars represent variable durations for touch and turn episodes across sessions and rats. Fig 3B shows hippocampal LFP aligned to the time of first whisker contact for a randomly selected set of trials. By visual inspection, a theta rhythm of about 10 Hz is evident around contact time. To better quantify the time course of LFP modulation through one trial, we measured the power spectrogram for the period extending 3 s on either side of first whisker contact. Fig 3C shows the LFP power spectrogram of the same session, averaged across trials, and normalized to the power at 4 Hz during the baseline interval (from 2.5 to 3 s before first contact). The spectrogram shows that the distribution of power across frequencies was related to the rat’s actions, as previously reported [23]). The theta band showed a marked increase in power late in the approach, remained high during touch, and dropped suddenly as the rat turned to the reward spout; theta power fell below baseline levels during reward consumption.

Next we considered the LFP power spectrum separately for the four behavioral episodes—baseline, approach, touch, and reward (Fig 3D). In all episodes, the peak of the LFP power spectrum occurred within the theta range of 5–12 Hz [5]). At baseline, the peak frequency of theta rhythm was 7.87 ± 0.25 Hz (*n* = 4 rats). Peak frequency was slightly higher during approach (8.25 ± 0.29 Hz, *n* = 4 rats) and touch (8.75 ± 0.5 Hz) although only during touch was the peak frequency significantly higher than during baseline. Reward episodes showed a significantly lower peak frequency (7.25 ± 0.28 Hz) compared to baseline, approach, and touch (all comparisons using one-way ANOVA *p* = 0.0004, with multiple comparison test). Moreover, mean theta power during touch and approach was significantly higher than during baseline and reward periods (randomization test for each rat, with Bonferroni correction, *p* < 0.01; see Materials and Methods). We hypothesize that, during approach, animals began to attend to incoming signals from their whiskers in preparation for the texture discrimination; the LFP changes might reflect an engagement of the hippocampus in the rhythm of the sensorimotor system in order to optimize the intracortical transfer of relevant sensory inputs. Fig 3C shows that task events also modulated the LFP spectrogram in other frequency ranges (e.g., from 20–40 Hz); however, this report focuses only on the theta range.

We also computed the LFP power spectrum when the rats were positioned in an open arena; this served as a control condition because the rats, whether resting (immobile and whisking) or active (walking and whisking), did not need to identify tactile objects or to compare incoming signals to a memorized spatial rule, as they did during the texture task. The peak frequency of theta rhythm as rats walked (8.50 ± 0.01 Hz, *n* = 2 rats) was higher than during rest episodes (7.80 ± 0.35 Hz, paired *t* test, *p* < 0.05), but there was no significant difference between theta peak frequency during walking versus that during the touch epoch of the texture discrimination task (8.75 ± 0.5 Hz, *t* test, *p* = 0.54). Moreover, theta power was higher during walking episodes than it was during rest and touch episodes (randomization test with Bonferroni correction, *p* < 0.01 for each rat, Fig 3E), confirming that movement through the environment is accompanied by increased theta rhythm frequency and power [25,26].

An issue of interest was the distribution of whisking frequency during the tactile task (Fig 3F). During approach to the texture, whisking frequency was broadly distributed between 6 and 12 Hz. During actual texture contact, whisking frequency was concentrated in a narrower band with peak at 10 Hz (*n* = 4 rats). Moreover, whisking power during touch was higher than during approach (randomization test with Bonferroni correction, *p* < 0.01 for each rat).

### Task Dependence of the Interaction between Hippocampal Theta Rhythm and Whisking

**Fig 4:**
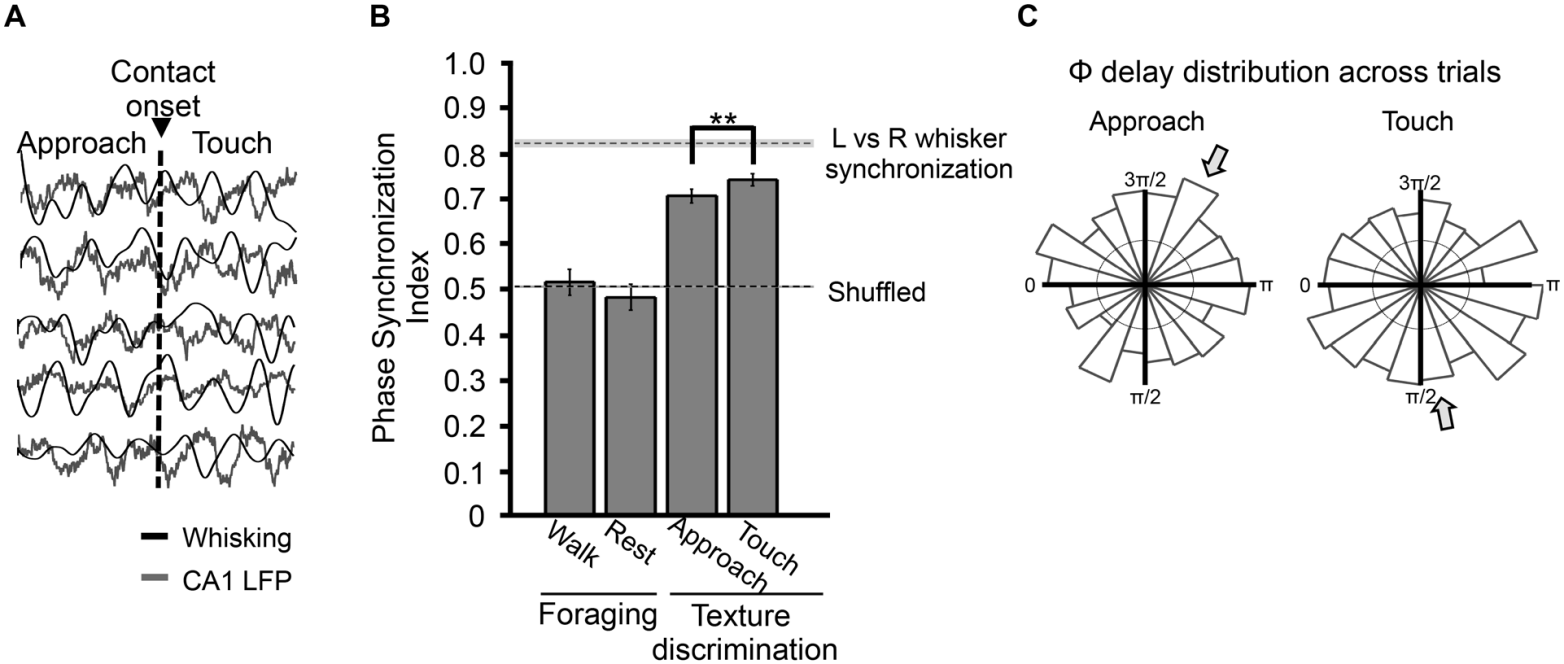
Whisking-theta synchronization during tactile discrimination task and foraging. **(A)** Simultaneous traces of whisking oscillation and theta rhythm aligned to contact onset for five trials randomly chosen from one session. **(B)** Phase Synchronization Index (PSI) during walk and rest episodes while foraging (*n* = 2 rats, 163 and 175 trials, respectively), and during approach and touch episodes during the texture discrimination task (*n* = 4 rats, 915 trials). Values correspond to mean ± 95% confidence interval. Dashed lines represent lower and upper limit of PSI values; lower limit (PSI = 0.507) is the mean + 95% confidence interval PSI of random sets built by shuffling rats and conditions. Top dashed line corresponds to the upper limit for PSI based on the coherence between whisking of the left versus right sides of the snout (PSI = 0.82, *n* = 4 rats, 1052 trials). Conditions were compared by applying a two-sample randomization test, *p* < 0.0001; see Materials and Methods. **(C)** Polar plots of theta-whisking phase delay distribution during approach and touch, sampled from correct trials showing a significant degree of synchronization (trials exceeding 95% confident limit). The Rayleigh test was applied to test for phase delay preference across trials. During approach the test showed no significant clustering (Rayleigh test, *p* = 0.06), while during touch the mean phase delay became clustered across trials (Rayleigh test *p* = 0.04). The circle centered at the origin represents 20 trials. All data are available at http://figshare.com/s/99b31b8a567f11e5b81d06ec4bbcf141.

A previous study [21] in which rats explored a runway in dimmed light did not find significant phase coherence between hippocampal theta rhythm and whisking; however, the authors allowed that “…it is possible that the theta rhythm and whisking will phase lock under certain circumstances, such as when a rat learns to discriminate an object with the vibrissas, as opposed to whisk in air” (p. 6,522), a prediction reiterated later [27]. The present behavioral task involved associating incoming sensory signals with a stored spatial rule—the reward location associated with that stimulus—a process that engages the hippocampus [23]. We hypothesize that the coherence between CA1 theta and the whisking rhythm might depend on behavioral context; more precisely, coherence could emerge during whisker-mediated texture identification. We tested this hypothesis by measuring the Phase Synchronization Index (PSI) between whisking cycles and hippocampal LFP when rats performed the discrimination task versus when they walked through the open arena, a condition in which signals from the whiskers had no explicit connection to information stored in hippocampus. PSI captures the degree of phase synchronization in short time windows without amplitude confounds (see Materials and Methods). Fig 4A illustrates five samples of simultaneously recorded whisking and hippocampal LFP recorded during the discrimination task, aligned to contact onset.

Whisking-to-hippocampal LFP phase synchronization during the tactile discrimination task was highly significant in the four rats examined (Fig 4B). The observed PSI averaged across rats was 0.71 during approach to the texture and 0.74 during touch (915 approach and touch episodes). Both values were above the upper bound of the 95% confidence limit (PSI = 0.507, labeled “shuffled” in Fig 4B) derived through the bootstrap method (see Materials and Methods). The PSI was significantly higher during touch than during approach (*n* = 915 paired episodes, two-sample randomization test, *p* < 0.0001).

It is informative to compare the observed coherence values to an estimate for their upper limit—the average coherence between the whisking motion of left and right whisker pad, a value of 0.82. During the approach phase and the touch phase, coherence was on average 64% and 74% as large, respectively, as its upper limit (i.e., proportion of the distance from chance level to the upper limit).

In contrast, as the animals foraged in the open arena, the level of coherence compared to that derived from the bootstrap value was reduced during rest (*n* = 163 episodes; PSI = 0.48, *p* = 0.03) and was unaltered during walking (*n* = 175 episodes; PSI = 0.52, *p* = 0.79). Absence of coherence could not be due to theta phase resolution, as theta power during foraging was as high as during the texture discrimination task (Fig 3E). The average PSI during approach and touch was 46% greater than during foraging. In sum, when the rat approached and contacted the stimulus in the context of the tactile classification task, the level of coherence between hippocampal theta and whisking increased markedly with respect to the chance level and to that observed in the control condition.

We examined the phase relationship between whisking and hippocampal theta during approach and touch. Fig 4C (upper panel) shows the phase delay (Φ) distribution in polar coordinates taken from all correct trials with significant value of coherence. Both during approach and during touch, the theta-whisking phase delay showed a borderline-significant degree of clustering (approach: *n* = 714, Rayleigh test, *p* = 0.06; touch: *n* = 776, Rayleigh test, *p* = 0.04). Though the phase delay was widely distributed across trials, the mean values are indicated by arrows: 4.25 radians during approach and 1.63 radians during touch.

### Does Theta-Whisking Coherence Predict Performance?

We posited that the efficiency of integration of stimulus information into memory and decision making centers is augmented when the rat’s sensorimotor rhythms are coherent with central oscillations, including that of the hippocampus. The prediction ensues that the likelihood of an incorrect choice would be greater on trials with low theta-whisking synchronization. Although an error could originate at any stage of processing, we hypothesize that some proportion of incorrect trials arose as a consequence of reduced theta-whisking synchronization. Consistent with the prediction, on incorrect trials mean PSI during the touch interval was 0.69, significantly lower (two-sample randomization test, 10,000 iterations, *p* = 0.0018) than the PSI of 0.74 on correct trials, when controlled for touch duration (touch duration of correct versus incorrect trials, *t* test, *p* > 0.05). The distribution of PSI values was more dispersed on incorrect trials, in accordance with the idea that not all errors happened for reasons related to the integration of sensory information into hippocampus.

**Fig 5:**
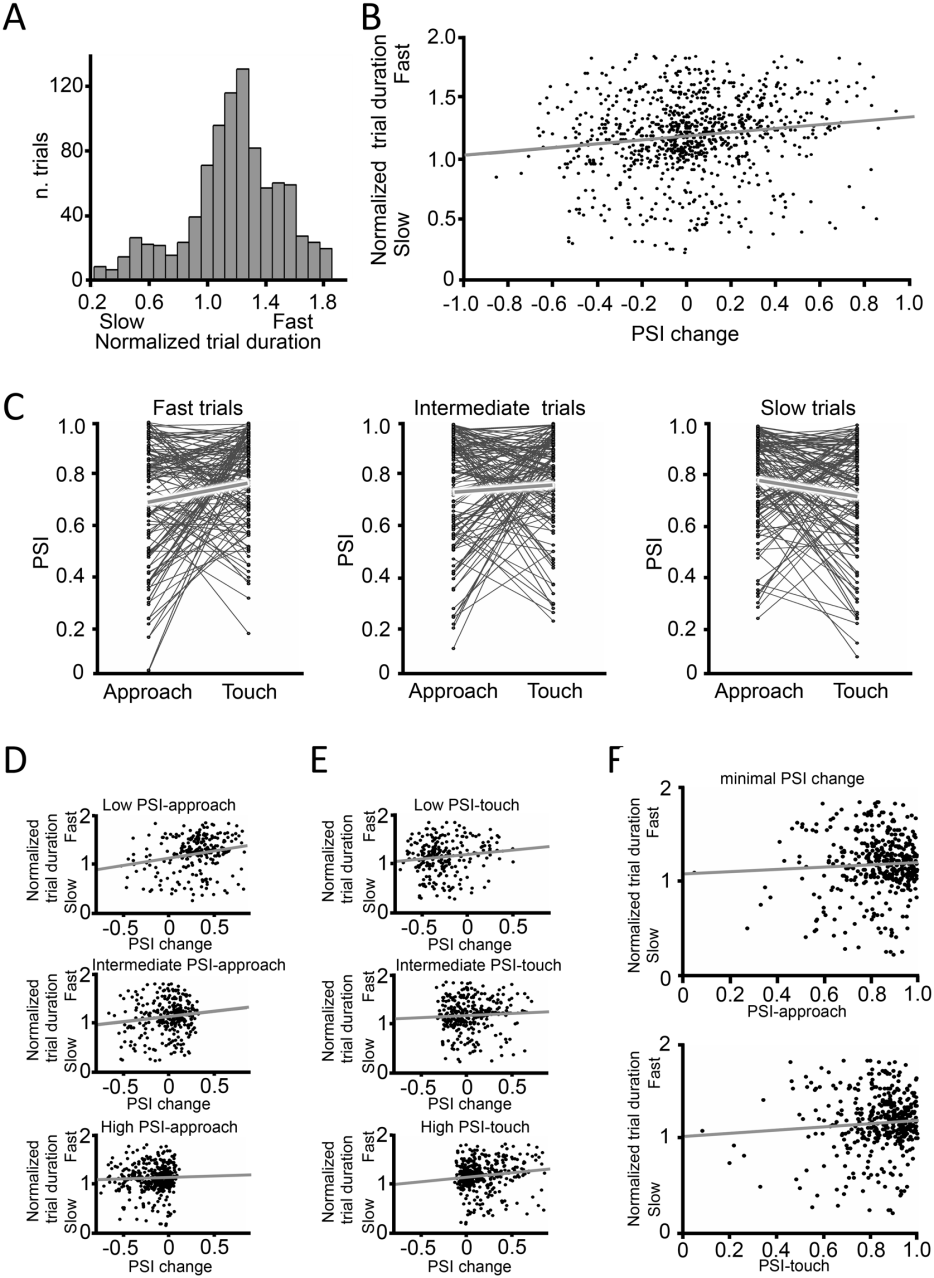
Touch duration and whisking-theta phase synchrony. **(A)** Normalized duration distribution of all trials. The time elapsed from first touch until withdrawal on each trial was normalized by subtracting it from the maximum value for that rat. **(B)** Correlation between approach-to-touch PSI change (= PSI-touch—PSI-approach) and normalized duration. Points are 915 individual trials. The fitted line is the least squares linear regression. **(C)** within-trial paired PSI-approach and PSI-touch values for slow, intermediate, and fast trials. To form the groups, mean and standard deviation (SD) of trial duration per rat was calculated. Slow trials were those from mean duration + 0.4*SD to 2 s. Intermediate trials were from mean duration − 0.4*SD to mean duration + 0.4*SD. Fast trials were those from 0.15 s to mean duration − 0.4*SD. We excluded trials in which duration was shorter than 0.15 s or longer than 2 s (86 trials) because such outliers may reflect an altered strategy. Average PSI change in each plot is shown as thick black segment. Only 50% of all trials (randomly selected) are illustrated to avoid clutter. **(D)** Correlation between PSI change (PSI-touch—PSI-approach) and normalized duration conditional on PSI-approach. Mean and SD of PSI-approach was calculated and trials were divided in groups. Low trials were those with PSI-approach < mean − 0.5*SD, intermediate trials were those with PSI-approach within +/- 0.5*SD of the mean, high trials were those with PSI-approach > mean + 0.5*SD. Correlation coefficients and their significance are given in the main text. **(E)** Correlation between PSI change and normalized duration conditional on PSI-touch. Low trials were those with PSI-touch < mean − 0.5*SD, intermediate trials were those within +/- 0.5*SD of the mean, high trials were those with PSI-touch > mean + 0.5*SD. Correlation coefficients and their significance are given in the main text. **(F)** Upper plot: correlation between PSI-approach and normalized duration in trials with minimal PSI change. Lower plot: correlation between PSI-touch and normalized duration in trials with minimal PSI change. For both analyses, statistics are given in the main text. All data are available at http://figshare.com/s/99b31b8a567f11e5b81d06ec4bbcf141.

Our view of the functional role of sensorimotor/hippocampal coherence also led us to examine the relationship between PSI and trial duration (the inverse of trial speed), measured as the time elapsed between first texture contact and withdrawal from the stimulus. We created a normalized trial duration measure such that a speedier trial gave a higher value, as follows: normalized trial duration = maximum trial duration for that rat − single-trial touch duration. The distribution of normalized trial duration values for all rats and all sessions is plotted in Fig 5A. On correct trials (915 trials, four rats), as expected, the value of PSI-touch was significantly and *positively* correlated with trial speed (Pearson correlation, r = 0.136 and *p* = 3.8*10^−5^). Unexpectedly, however, on these same correct trials the value of PSI-approach was *negatively* correlated with trial speed (Pearson correlation, r = -0.068 and *p* = 0.040). From these seemingly contradictory findings, we were drawn to consider an alternative sequence of events that might encompass both the approach and touch PSI observations: we asked whether the *change* in value of PSI from approach to touch (PSI-touch—PSI-approach) might be a more robust predictor of task performance than PSI-touch or PSI-approach taken separately. The chain of events could be as follows. Initiation of the approach toward the stimulus entails an increase in hippocampal theta power and phase-coherent whisking. Later, the afferent sensory volley arising from whisker contact with the plate acts as a timing signal to sharpen the sensorimotor/hippocampal synchronization and thus facilitates hippocampal processing of texture information; however, the timing signal is effective only on those trials in which hippocampus is receptive to phase modulation. In contrast, when the hippocampal oscillation is less receptive to the timing signal, sensorimotor/hippocampal synchronization upon contact may remain constant or even decline, thus slowing the execution of the task.

As a first test, we looked for a correlation between within-trial PSI change (= PSI-touch—PSI-approach) and normalized trial duration. Fig 5B demonstrates that the change in PSI from approach to touch was significantly and positively correlated with faster trial execution (Pearson correlation, r = 0.146, *p* < 0.0001, slope of linear fit = 0.16).

Next, we divided the set of trials of each rat into three groups, according to touch duration: fast, intermediate, and slow (see Fig 5 legend for details). Fig 5C presents the paired PSI-approach and PSI-touch values for all trials of each group. Fast trials were characterized by an *increase* in PSI from approach to touch (*n* = 329, *p* < 0.001), while slow trials were characterized, on average, by a *decrease* in PSI from approach to touch (Wilcoxon signed rank test, *n* = 258, *p* = 0.0017). Intermediate trials showed no change in PSI (*n* = 328, *p* = 0.64). Thus, on trials in which texture contact led to an increase in sensorimotor/hippocampal phase synchronization, rats turned toward the reward spout earlier, suggesting that they could convert sensory input to decision more rapidly.

To confirm that PSI change was a more robust determinant of trial speed than were PSI-approach or PSI-touch alone, we grouped trials according to PSI-approach (low, intermediate, high; details in figure legend) and according to PSI-touch (low, intermediate, high). PSI change predicted trial speed (Fig 5D) whether PSI-approach alone was low (upper panel) or intermediate (middle panel) (Pearson correlation for low group: *n* = 248, r = 0.233, *p* = 2.16*10^−4^; for intermediate group: *n* = 290, r = 0.129, *p* = 0.027). However, when PSI-approach was high (lower panel), there was no significant effect of PSI change (*n* = 377, r = 0.043, *p* = 0.403). This result can be attributed to the scarcity of trials in which a high PSI-approach was followed by a positive PSI change—the scatter plot reveals PSI change truncated just above 0 and all points clustered to the left. There may be a PSI-approach threshold above which the tactile contact volley can provide no additional timing signal and hence no further boost in synchronization between the sensorimotor system and hippocampus. Likewise, PSI change predicted trial speed (Fig 5E), whether PSI-touch was low or high (Pearson correlation for low group: *n* = 232, r = 0.136, *p* = 0.037; for high group: *n* = 380, r = 0.133, *p* = 0.0091). In the case of intermediate PSI-touch, PSI change was not well-correlated with trial duration (*n* = 303, r = 0.0591, *p* = 0.301). Taken together, these data suggest that even when conditional on restricted ranges of PSI-approach and PSI-touch, PSI change remains a predictor of the rat’s efficiency in collecting sensory data and acting upon its choice.

As an additional test of the hypothesis that approach-to-touch PSI change was a primary factor underlying the rat’s performance, we selected trials in which PSI change was minimal (-0.15 < PSI change < 0.15). If PSI change predicts trial speed, then among these trials variation in PSI-approach (Fig 5F, upper plot) and variation in PSI-touch (Fig 5F, lower plot) should have little additional effect. As predicted, neither showed significant correlation with speed (Pearson correlation, PSI-approach: *n* = 405, r = 0.0517, *p* = 0.299. PSI-touch: *n* = 405, r = 0.075, *p* = 0.128).

**Fig 6:**
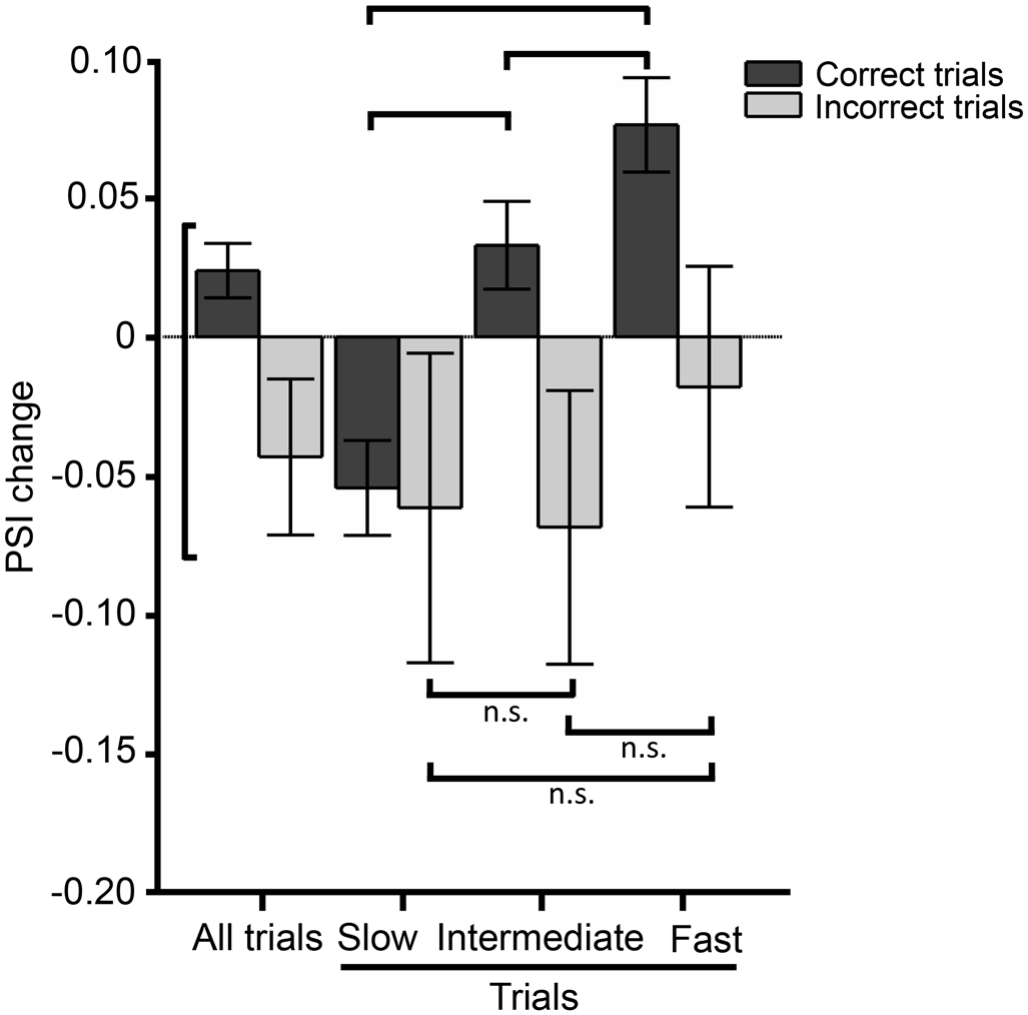
PSI change on correct versus incorrect trials. Comparison between PSI change for correct and incorrect trials. Left plot: all trials. Additional plots: similar to Fig 5C, trials were divided into three groups—slow, intermediate and fast—based on the touch duration. Mean values of PSI change are plotted for each group. Error bars are standard error of the mean. Black lines indicate one-tail *t* test comparison between groups, * indicates *p* < 0.05, n.s. indicates *p* > 0.05. All data are available at http://figshare.com/s/99b31b8a567f11e5b81d06ec4bbcf141.

The analysis above considered only correct trials. A further prediction is that PSI change, from approach to touch, may be lower or perhaps negative on error trials; this is confirmed in Fig 6, which shows that PSI change was 0.024 averaged across all correct trials and -0.043 averaged across all incorrect trials (correct versus incorrect PSI change, one-tail *t* test, *p* = 0.0073). Next, we measured the PSI change values on correct and incorrect trials for three groups based on trial duration: slow, intermediate, and fast. Confirming the results of Fig 5C, on correct trials mean PSI change was negative, near zero, and positive on slow, intermediate, and fast trials, respectively (Fig 6). Statistical details are given in the figure legend. On incorrect trials PSI change tended to be negative, with no significant variation according to trial duration. This is consistent with the notion that poorer integration of sensory signals into hippocampus occurred when vibrissal contact with the stimulus failed to evoke a boost in coherence between the sensorimotor system and hippocampus, leading to slower execution of the behavior and even to errors.

In conclusion, numerous analyses indicate that the efficiency with which the rats integrated sensory signals and converted the percept into an action was related to the magnitude of change in coherence between the sensorimotor systems and the hippocampus evoked by the rats’ contact with the stimulus.

**Fig 7:**
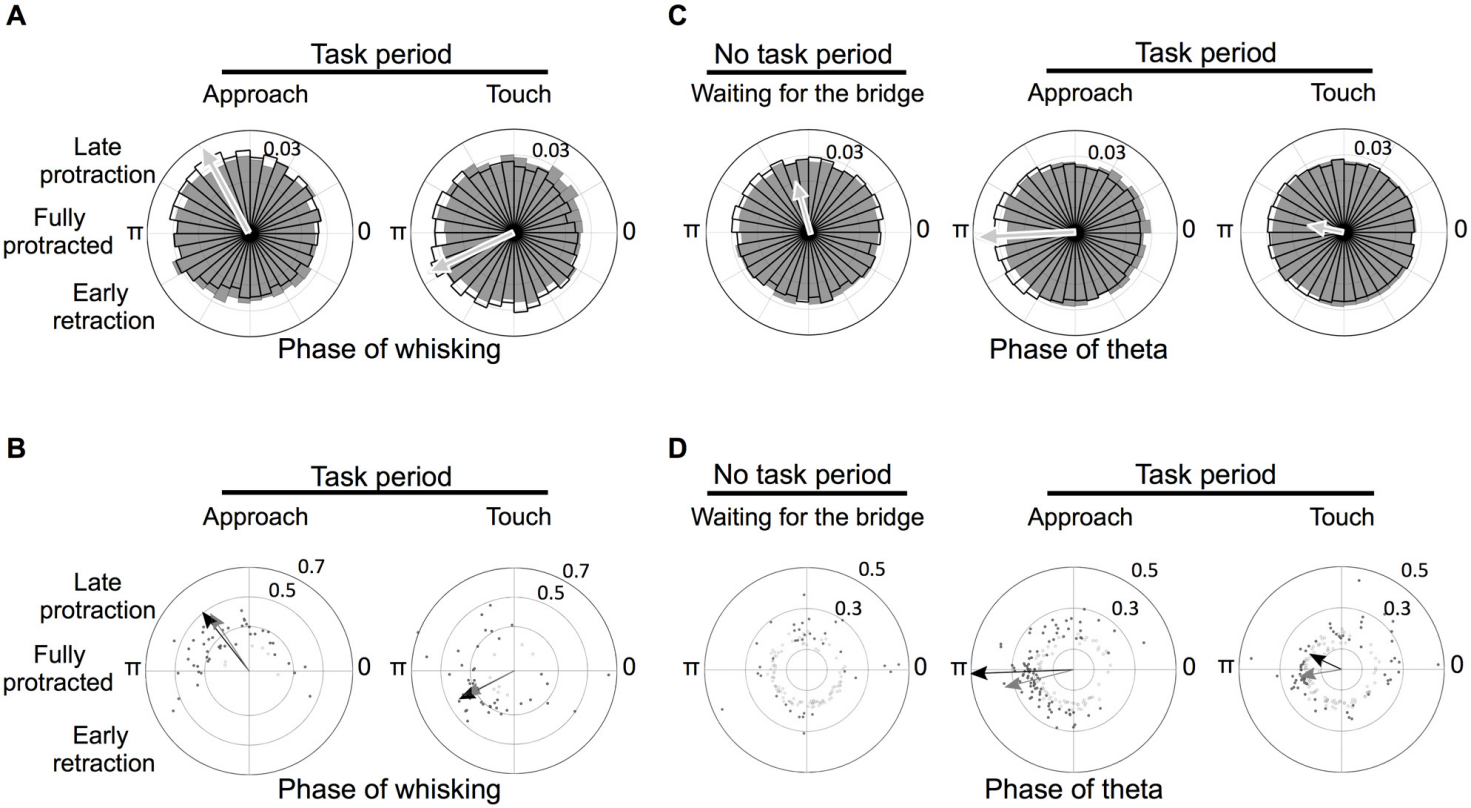
Phase-coherence between barrel cortex, whisking cycle, and theta. **(A)** Angle histogram of the phase distribution of all spikes fired by significantly phase-coherent neurons in relation to the whisking cycle during approach (16,707 out of 20,868 spikes [80%]) and touch (17,058 out of 22,467 spikes used [76%]). The radial axis indicates the spiking probability for the corresponding direction (bin width: 10 degrees). The value 0.03 marks the axis corresponding to the probability value of 3%. (**B)** Mean preferred phase of each studied neuron. **(C)** Angle histogram of the phase distribution of all the spikes fired by significantly phase-coherent neurons in relation to theta (no task period: 26,650 out of 144,912 spikes used [18%], approach: 105,808 out of 168,178 spikes used [63%], touch: 78,030 out of 180,009 spikes used [43%]). (**D**) Mean preferred phase of each neuron in relation to theta. All data are available at http://figshare.com/s/99b31b8a567f11e5b81d06ec4bbcf141.

Having evaluated the relation between the whisking rhythm and hippocampal theta, in the following sections we consider the coherence between barrel cortex and these same two rhythms. The analysis is organized as barrel cortex coherence with whisking (Fig 7A and 7B) and barrel cortex coherence with hippocampal theta (Fig 7C and 7D).

### Phase-Coherence between Barrel Cortex and the Whisking Cycle

We tested each barrel cortex neuron for non-uniformity in the phase distribution of its spikes with respect to the whisking cycle (Rayleigh test, *p* < 0.05). In accordance with previous studies [28,29]), the spiking of most barrel cortex neurons was phase-locked to whisking during the tactile discrimination task (approach: 45 out of 51 [88%], and touch: 43 out of 51 [84%]). To visualize phase relationships, we pooled all the spikes fired from the set of significantly phase locked neurons; in Fig 7A the black-outlined circle sectors represent the spikes’ distribution in relation to the whisking cycle during approach and touch. The radial extent of each sector indicates the proportion of all spikes that occupy that bin of the cycle (bin width: 10 degrees). The underlying gray bins indicate the phase angle histograms obtained when the phase relationship between spike occurrence and instantaneous whisking phase was shuffled across time, which effectively assigned one random phase value to each spike. The arrow in each plot indicates the circular mean phase of spiking, proportional in length to the concentration parameter κ from the von Mises distribution (see Materials and Methods). While, as expected, the shuffled spikes (gray) were distributed uniformly across the whole whisking cycle (Rayleigh test, *p* > 0.1), the spikes emitted during both approach and touch were significantly modulated in relation to whisking phase (Rayleigh test, *p* < 0.0001). The mean phase of spikes shifted from approach (2.09 radians) to touch (3.56 radians): during approach spikes were concentrated late in the phase of protraction, whereas during touch spikes were concentrated around the start of retraction. The phase distribution of spikes from the entire set of neurons (including those not significantly phase locked) yielded similar results (S1A Fig).

The pooling of spikes, as above, provides an estimate of the message received by an observer (target population of the brain) that integrates all incoming signals with equal weights, ignoring the identity of the neuron emitting the spike; neurons with higher firing rate would have a larger influence on the observer. Next we considered the same dataset but, instead of pooling spikes, we examined the phase preference of each neuron with respect to whisking. By doing this, we examined the phase entrainment of the whole population, giving equal weight to each neuron and neglecting the heterogeneity in the number of spikes fired by each neuron. Cell-by-cell averaging of neuronal responses has been proved to be an effective method to decode information from a population of neurons [30,31]). Fig 7B shows the mean preferred phase of each neuron (significantly phase locked neurons [Rayleigh test, *p* < 0.05] given by black delimited dots; non-significantly phase locked neurons by gray dots): angle denotes phase preference and distance from the origin denotes κ parameter. As was seen for pooled spikes (Fig 7A), the phase preferences of neurons significantly entrained to the whisking cycle were clustered around late protraction during approach (Rayleigh test: *p* < 0.000001), whereas during touch preferred phases were clustered around early retraction (Rayleigh test: *p* < 0.00001). Mean angles across neurons with significant phase locking (black arrows) during approach and touch were 2.24 radians and 3.62 radians, respectively, and thus well aligned with directions derived from all pooled spikes (Fig 7A). Arrows are proportional in length to the concentration of units around the mean phase value, where the maximum length is normalized to the radial axis, 0.5. In spite of the increase in firing rate for most units upon touch, the strength of phase locking, per neuron, did not change significantly (κ_approach_ = 0.339 ± 0.087, κ_touch_ = 0.348 ± 0.112, Wilcoxon rank sum test, *p* > 0.05, see Materials and Methods). Similar results were obtained when neurons without significant phase-locking were included in the analysis (gray arrows, Fig 7B). The whisking phase preference of all significantly phase-coherent neurons is also illustrated by the phase angle histogram in Supporting Information (S2A Fig).

### Phase-Coherence between Barrel Cortex and Hippocampal Theta

Since coherence between hippocampal theta and whisking were significantly higher during tactile discrimination than during foraging (Fig 4), we expected barrel cortex units to be increasingly phase locked to hippocampal theta during the discrimination task. We explored phase relationships between barrel cortex and theta by the same procedures described above. Since this measurement did not rely on high-speed video of the whiskers, we could extend the analysis to the pre-trial onset epoch, as the rats waited for the foot bridge and texture plate to be presented (waiting period). By direct observation, we noted that they actively whisked over the edges of the platform to detect the positioning of the bridge.

First we tested each barrel cortex neuron for non-uniformity in the phase distribution of its spikes with respect to hippocampal theta (Rayleigh test, *p* < 0.05). Though the concentration of single cell’s preferred phases did not change from the waiting period (*n* = 136, κ = 0.242 ± 0.080) to the approach (*n* = 161, κ = 0.235 ± 0.064) and touch (*n* = 163, κ = 0.238 ± 0.088, Kruskal-Wallis test, *p* > 0.05), a larger proportion of barrel cortex neurons became phase locked to hippocampal theta, around their mean preferred phase. In the pre-trial period, 37 out of 136 barrel cortex neurons (27%) were significantly coherent with theta. Once the bridge was in place, rats approached the texture and phase coherence with theta increased significantly: 92 out of 161 neurons (57%) were coherent. During the touch epoch, the proportion of neurons coherent with theta decreased to 64 out of 163 (39%). In summary, during approach, the proportion of neurons phase-coherent with hippocampal theta exceeded the proportions during the waiting period (two-proportion z-test, z = 5.1858, *p* = 0) and the touch period (z = 3.2204, *p* = 0.001). The percent of neurons with theta phase locking might have diminished from approach to touch because spike times were dictated less by internal brain dynamics and more by vibrissal kinematic events [32], which could be distributed along both protraction and retraction. Likewise, a possible explanation for the preferred phase change is that as retraction began, neuronal firing was evoked by the translation of the whiskers along the textured surface.

To examine phase coherence more directly, all the spikes fired by the significantly phase-locked neurons in each epoch were pooled and plotted in relation to the theta cycle. The results are given as phase angle histograms during waiting, approach, and touch in Fig 7C. The black-outlined circle sectors represent the spikes’ distribution in relation to the theta cycle during waiting, approach, and touch. The radial extent of each sector indicates the proportion of all spikes that occupy that bin of the cycle (bin width: 10 degrees). The underlying gray bins indicate the phase angle histograms obtained when the phase relationship between spike occurrence and instantaneous theta phase was shuffled across time. As expected, spiking in the shuffled data was distributed uniformly across the whole theta cycle (gray bins, Rayleigh test, *p* = 0.8). All three conditions yielded significant modulation of spiking phase (Rayleigh test, *p* < 0.00001), although during approach the modulation was strongest. The arrow in each plot indicates the circular mean phase of spiking, proportional in length to the concentration parameter κ from the von Mises distribution. The mean preferred phase during waiting, approach, and touch were 1.80, 3.18, and 2.94 radians, respectively. The phase distribution of spikes from the entire set of neurons (including those not significantly phase locked) yielded similar results (S1B Fig).

Next we considered the same dataset but, instead of pooling spikes, we examined the phase preference of each neuron with respect to theta. Fig 7D shows the mean preferred phase of each neuron) during pre-trial waiting, approach, and touch (significantly phase locked neurons (Rayleigh test, *p* < 0.05) given by black delimited dots; non-significantly phase locked neurons by gray dots): angle denotes phase preference and distance from the origin denotes κ parameter. Only approach and touch yielded significant clustering of neurons’ preferred theta phase of spiking (Rayleigh test, *p* = 0.097 during pre-trial waiting, *p* < 0.00001 during approach, *p* < 0.05 for touch). Mean direction across neurons with significant phase locking during approach and touch, given by black arrows (3.18 and 2.70 radians, respectively), was in accordance with those derived from the analysis of all spikes (Fig 7C). Length of the arrows, similar to Fig 7B, is proportional to the concentration of units around the mean direction. Similar results were obtained when neurons without significant phase-locking were included in the analysis (gray arrows, Fig 7D). Arrows are not given in the left plot (waiting for bridge) because preferred theta phase of spiking was not significant. The theta phase preference of all significantly phase-coherent neurons is also illustrated by the phase angle histogram in Supporting Information (S2B Fig).

## Discussion

As early as 1970, it was noted that in exploring rats, the whisking rhythm (measured by electromyiography [EMG]) could be briefly synchronized with the theta rhythm, measured by electroencephalogram (EEG) recordings from the “limbic-hypothalamic system” [20]. In hamsters, during exposure to novel odorants, bouts of entrainment between sniffing, whisking, and hippocampal theta were observed [33]. However, the proposal of a direct temporal relationship between whisking and hippocampal theta rhythm was not supported by a study that assessed their coherence as rats foraged in a familiar open arena [21]. To resolve these apparent discrepancies, we designed a study in which rats associated sensory signals acquired by whisking with a stored behavioral rule—a process that engages the hippocampus [23]. Coherence between the CA1 theta rhythm and whisking rhythm emerged specifically and selectively during whisker-mediated stimulus identification such that phase synchronization increased by over 40% during approach and texture palpation (Fig 4B). Moreover, in the transition from approach to touch, an increase in coherence between hippocampal theta and whisking was correlated with faster and more accurate sensory integration (Fig 5). Taken together, these results support the proposal that the entrainment of sensorimotor systems with hippocampus, at the theta frequency, plays a role in transmitting sensory information into memory and decision-making centers. Moreover, the afferent sensory volley arising from whisker contact with the plate might act as a timing signal to sharpen the sensorimotor/hippocampal synchronization and thus increase the efficiency of execution of the task.

Upon contact with the plate, the preferred phase of the relationship between whisking and hippocampal theta (Fig 4C) shifted from 4.25 radians (approach) to 1.63 radians (touch), suggesting that sensory signal onset reaches the hippocampal structures and modulates the phase of the theta rhythm [34]. The phase relationship between hippocampal theta and the firing of single neurons in barrel cortex also varied according to the task: a greater proportion of neurons was phase locked to theta when rats were engaged in the discrimination task compared to the period when rats waited for the foot bridge and texture plate to be presented (Fig 7D).

### Time Course of Interaction between Theta Rhythm and Whisking

Each trial began when the foot bridge rotated into the position whereby the rat could perch on it and approach the texture plate. Coherence between whisking and hippocampal theta augmented as the rat leaned forward (“approach”), about 200–300ms before whisker contact occurred (Fig 4B and 4C). In the dark, the noise produced by the motor that moved the bridge was a predictor of forthcoming stimulus availability. We suggest that external sensory inputs such as the motor sound, together with proprioceptive inputs such as leaning and reaching, triggered synchronization between sensory and memory structures. Before making their choice, rats typically make just two to four whisks upon the texture for a total duration of about 500 ms (Fig 1; also see [35–37]). For this reason, preparatory entrainment would be important in permitting even the first whisker-to-discriminanda touch to be optimally processed. During texture palpation, whisking–theta synchronization increased with respect to the approach episode, indicating that coherence was also affected by extrinsic signals, namely, contact with the plate; a larger increase in synchronization predicted a faster correct trial (Fig 5) and failure to increase synchronization was on average more associated with incorrect trials (Fig 6). This increase was accompanied by other changes: (i) CA1 theta peak frequency increased with respect to baseline and approach (Fig 3D), (ii) whisking frequency became concentrated in a narrower band with peak at 10 Hz (Fig 3F), and (iii) the phase difference between theta and whisking shifted and mean phase delay of most trials became as small as a quarter cycle (Fig 4C). The increase in peak frequency of the theta rhythm (from 7.87 ± 0.25 Hz at baseline to 8.75 ± 0.5 Hz during touch) is the first finding, to our knowledge, of a rise in theta frequency related to sensory sampling in the context of a memory task. In contrast, earlier studies showed a downward shift in theta frequency during novelty detection and odor sampling onset in the learning phase [38,39]. Hippocampal theta is known to increase in power and frequency during locomotion through an environment, as compared to inactivity [26,40]. Inasmuch as the rats in our task moved only a few cm as they reached forward and sampled the stimulus, locomotion is not the best explanation for the increase in theta power and frequency.

It has been suggested that the triggering of phase reset of hippocampal theta band by stimulus onset [41–43], may ensure that sensory input is integrated at an optimal phase of the oscillation. This may have important implications for memory-related mechanisms that are associated with a specific theta phase [42,44–46]). For two independent oscillators, phase coherence can be achieved by resetting the phase of just one oscillator. In our dataset, we found indications consistent with hippocampal phase resetting by touch onset; however, the number of trials available was not sufficient to support robust statistical testing. Yet, the shift in preferred phase between theta and whisking at the junction from approach to touch (Fig 4C) is in line with these observations.

### Barrel Cortex Activity in Relation to Behavior

During approach, most barrel cortex neurons tended to spike late in the protraction phase of the whisking cycle (Fig 7A and 7B), consistent with earlier studies of rats whisking in the air (reviewed by [47]; one study, however, found no marked phase preference [28]. During touch, preferred phases were clustered around the start of whisker retraction (Fig 7A and 7B), the instant when whisker kinematic events are most likely to evoke spikes [37]. Moreover, because the timing of neuronal spikes in hippocampus is entrained to theta [48], theta could act to organize the routing of information during sensory processing and memory retrieval [49,50]. Thus, the entrainment to theta of multiple brain regions [6,14,51]) would augment the temporal precision by which neurons from downstream areas (higher sensory and association areas) would process rhythmic primary somatosensory spiking activity. In moments in which the rat’s attention is not directed to the identity of objects contacted through whisking, hippocampal theta may be coherent with non-tactile sensory systems.

### Brain Oscillations and Information Processing

Recent work has suggested that cognitive operations including memory [6,14,52], perception [53]), and decision making [50,54] are mediated by rhythmic modulation of cortical local field potential (LFP). If synchronization between oscillators plays a functional role in information processing, the animal’s behavior must reflect variations in synchronization. In a spatial memory task, the degree of coordination between prefrontal cortex and hippocampus theta rhythms was correlated with behavioral performance [6]). During performing a Y-maze rule-learning task, theta coherence between prefrontal cortex and hippocampus was significantly higher after animals acquired the rule [14]. Moreover, trials with high coherence were, on average, associated with higher levels of performance. The degree of synchronization of the olfactory bulb and hippocampal theta rhythms in an odor-discrimination task was positively correlated with performance [55], suggesting a functional significance of synchronized oscillation not only within widely distributed, multimodal spatial navigation systems but also within individual sensory systems.

Still, the generality of coupling brain regions by coherent oscillation remains to be demonstrated. Many species, including rats, mice, flying squirrels, gerbils, chinchillas, hamsters, shrews, porcupines, and opossums whisk, yet their theta frequency bands vary much less than do their whisking frequency bands [56]. The coherence between whisking and theta might be an incidence in a particular species, under particular conditions. Furthermore, whisking is correlated with nose, head, and sniffing movements in some but not in other species [57]. Overall, there is not adequate evidence in the literature to specify to what extent the brain’s multiple sensory and motor systems are entrained to theta.

### Directionality of Entrainment

Our finding of augmented PSI between the sensorimotor plant (expressed through the whisking oscillation) and the hippocampus during approach and touch raises the question of whether the sensorimotor system entrains hippocampal theta, or vice versa. Early lesion studies [18] showed that whisking is generated independently from the septo-hippocampal system; instead, the intermediate band of the ventral intermediate reticular formation (vIRt) is critical in generating the whisking pattern [58]. vIRt functions as the premotor pattern generator for rhythmic whisking and is part of a larger circuit devoted to inspiration (pre-Bötzinger complex). The pattern generator is situated within nested, closed loops [59,60] that, as a whole, mediate vibrissae-based sensation and motor control.

Hippocampal theta has been recorded in immobile rats prior to the initiation of lateral dodging movements in response to conspecific rats attempting to steal their food [61]. Following infusion of atropine into the medial septum, theta recorded during immobility is abolished and the rats are severely impaired at initiating movements. Thus, the trigger to the rat’s goal-directed actions (reaching forward and palpating the texture) might originate within the hippocampal complex and associated nuclei. However, once the task is initiated, sensory inputs may provide positive feedback: nucleus reticularis pontis oralis (RPO) and peduculopontine tegmental nucleus (PPT)—brainstem regions involved in the regulation of cortical arousal—receive somatosensory input [62,63] and have direct and indirect projections to the medial septal region of the hippocampal formation [61]. Electrical stimulation of pathways from RPO and PPT elicits theta oscillations in the hippocampal formation [61].

Based on the work summarized above, we suggest that the entrainment between hippocampus and the sensorimotor system may proceed by a sequence of events that includes the following elements: Hippocampal theta power increases prominently as the rat leans forward during approach (Fig 3C). The boost in hippocampal theta facilitates the concentration of whisking power in the theta band (note the change in whisking power from approach to touch in Fig 3F). Sensory inputs generated by contact of the whiskers provide positive feedback to the generation of hippocampal theta through the ascending brainstem pathways. The state of the hippocampal networks at the onset of approach and touch could predispose the system towards more or less efficient phase synchronization; we found that on trials when phase synchronization between whiskers and hippocampus increased upon touch, the trial was executed more efficiently and also there was a higher chance for the rat to perform correctly. In summary, we argue that there is no single direction of entrainment, but a reciprocal interplay in both directions.

The present work confirmed earlier notions and added several insights to existing knowledge. Our study emphasizes the task-dependence of coherence: hippocampal theta rhythm was coherent with the vibrissal sensorimotor rhythm only during moments when information about tactile object identity was integrated into the hippocampus. Rhythmic activity extended to the barrel cortex, where the spiking of most neurons became phase-locked to whisking as the rat approached the texture. High levels of coherence between the whisking rhythm and hippocampal theta were associated both with better performance and a more rapid response.

## Materials and Methods

### Subjects

Seven Wistar male rats (Harlan Italy, S. Pietro al Natisone, Italy) weighing about 300 g were housed in pairs and maintained on a 14/10-h dark/light cycle. All experiments were conducted during the dark phase of the daily cycle. Rats were habituated to handling for five days before experiments started. Food was offered ad lib throughout the experiment. Water was given during training as a reward and was also available ad lib for 10 min after training. The rats were under the care of a consulting veterinarian. Protocols followed the guidelines of EU Directive 2010/63/EU, established as Italian decree 26/2014, and were approved by the SISSA Ethics Committee and the Italian Ministry of Health license numbers 569/2015-PR and 570/2015-PR.

### Discrimination Task and Performance

Seven rats were trained to perform a tactile discrimination task [23]. The training setup consisted of a 10 cm x 30 cm elevated platform with two lateral water spouts, a movable bridge, and a motor that positioned one of the stimuli, in random order, in front of the platform just prior to trial onset (Fig 1A).

Four 10 (width) x 3 (height) cm textured Plexiglas rectangles, designated S1–S4, were used (Fig 1B) as stimuli. S1 was smooth. S2 was an irregular texture made by pressing P100 sandpaper into heat-softened Plexiglas. S3 and S4 were pieces of Plexiglas with vertical grooves of 1 mm width and depth cut at intervals of 2 mm (S3) and 4 mm (S4). Each stimulus was associated uniquely with one of the two lateral and opposite spouts (see Fig 1A for position of the spouts); rats learned to identify the texture plates and turn to the associated spout in order to receive a water drop as reward. If, after touching the plate, rats turned to the incorrect spout, no water was delivered and a timeout was given during the inter-trial interval before a next trial could take place. Only the first choice of the animal was considered as valid.

All rats except one were trained to discriminate two of the four textures; Rat 3 was trained to discriminate three textures, i.e., to associate two of the stimuli with one water spout and the third stimulus with the opposite spout. Associations between texture and reward location were fixed for each animal but were varied across rats (see Table 1 for stimulus/reward location pairings). Substitution of several different exemplars of S1–S4 ensured that rats did not use specific cues attached to one particular object. Moreover, potential olfactory cues were removed by cleaning with 75% alcohol at least once every session.

The rat self-initiated each trial by perching on the bridge and extending forward to contact the stimulus. Once it identified the texture and turned toward one of the spouts, the bridge retracted, signaling the start of the inter-trial interval (duration from 9 to 12 s). At the end of the inter-trial interval, the bridge came out again and the central motor positioned one of the stimuli in front of the platform, signaling the onset of the next trial.

Central and lateral light sensors were used to monitor the rat’s position so that its behavior could be synchronized with electrophysiological recordings and high-speed video. The central sensor, positioned directly in front of the texture plate, signaled the start (contact onset) and end of touch, whereas sensors positioned adjacent to the lateral spouts signaled the end of turn and start of reward consumption.

A high-speed video camera (Optronis CamRecord 450, Optronis) was positioned above the stimulus to capture whisker movements with 512 x 512 pixel-images at 1,000 frames per second (Fig 1C). The entire set up was commanded with LabVIEW software (National Instruments).

The rats’ performance in each recording session was above 70% correct (Fig 2A). To verify that this performance was above chance, we simulated the percent correct by randomly shuffling texture labels across trials. Repeating the shuffling procedure for 500 iterations provided a distribution of performance values that could be expected by chance. Compared to the chance distribution, performance of each rat exceeded a threshold of *p* < 10^−10^.

### Video Records and Whisker Tracking

Video clips during the discrimination task were recorded in a dim ambience using infrared light reflected in a circular mirror positioned under the rat; the head and whiskers were dark against a bright background. When the rat entered the field of view and approached the texture, it triggered the central sensor, which in turn triggered the video clip recording. The video clip lasted 750 ms and included the period from approach until withdrawal. We extracted whisker movements (mean angle of all detected whiskers in each frame) with the Standard Tracker free toolbox [24].

In addition to the discrimination task, two of the animals were also trained to walk across a 40 x 40 cm platform in which cereal flakes were delivered at random times and locations. A high-speed video recorder (Motionpro 2000; Redlake) was positioned above the platform to capture 800 x 600 pixel images of the whiskers at 200 frames per second as the rat foraged for a total of 30 min in each session (Rat 5: five sessions, Rat 6: eight sessions). The platform had a red glass floor to filter bright white light and was illuminated from below so that the animal’s head and whiskers were dark against a light background. In each session we collected an average of 150 seconds of high-speed video. Video segments were cut into pieces termed “trials” with duration of 500 ms, equal to the mean duration of the texture palpation episodes analyzed during the discrimination task. We took only trials where the animal was continuously whisking and extracted whisker movement manually because image contrast was below threshold for the automated tracking program. Manual tracking consisted of detecting the retraction/protraction start and end frame, and reconstructing the whisking cycle by modeling it as a sinusoid. Trials were divided into two groups according to the rat’s actions: “walk” and “rest.”

### Surgery and Recordings

Animals were anaesthetized with isofluorane or with a mixture of Zoletil (30 mg/kg) and Xylazine (5 mg/kg) delivered *i*.*p*. A craniotomy was made above left dorsal hippocampus and barrel cortex, centered 2.76 mm posterior to bregma and 3 mm lateral to midline [64]. A 12-tetrode microdrive (Neuralynx) was implanted (hippocampal bundle: -3 mm AP, -2 mm L; barrel cortex bundle: -2.7 mm AP, 5.5 mm L) and fixed by dental cement. Rats were given the antibiotic enrofloxacin (Baytril; 5 mg/kg delivered through the water bottle) and the analgesic caprofen (Rimadyl; 2.5 mg/kg, subcutaneous injection) for 1 wk after surgery. For 10 d after surgery, rats had unlimited access to water and food. Recording sessions in the apparatus began thereafter. Tetrodes were moved individually until they reached CA1 (DV: 3.12 mm) and barrel cortex layer 4–5 (DV: 0.9–1.1mm). To acquire action potentials, the signal was amplified (1,000–5,000 times), bandpass filtered (300–6,000 Hz), and digitized (32 kHz). Spikes were sorted offline using MClust [65,66]). Most electrodes yielded multiunit neuronal clusters, but in some cases, we could isolate single units with a pronounced refractory period. After the first encounter with neuronal populations at the desired depth, electrodes were advanced in steps of 40 um. Only neurons with consistent action potential shape and interspike interval histogram throughout the session were included in the analysis. Data recorded in different sessions from the same tetrode were always considered to be different clusters. LFP was obtained from CA1 by treating the raw signal with amplification (1,000–2,000 times), bandpass filtering (1–475 Hz), and digitization (2 kHz). The reference electrode was the stainless steel guide tube touching the surface of the brain, plus a tetrode located in white matter as local reference. Online visual inspection of prominent theta waveforms in addition to histology confirmed the position of the tetrodes (Fig 2C).

### Definition of Behavioral Episodes

Using optical position sensors and video analysis, we divided each trial into four episodes: (i) “approach” (the rat reached the platform edge, perched on foot rest, and leaned forward to reach the texture plate), (ii) “touch” (from onset of first stimulus contact to offset of final contact, provided by the central light sensor), (iii) “turn” (from offset of final stimulus contact until arrival at the reward spout, given by the lateral sensors), and (iv) “reward” (from arrival until departure from reward spout). Although well-trained rats carried out the task with stereotypical actions and there was no systematic difference across animals, the duration of these four episodes varied (Fig 3A). Spectral analyses required all epochs to be of equal length. To that end, we set 500 ms, close to the 540 ms mean value of the touch interval across rats and sessions, to be the standard duration. For the reward episode, we selected an interval of 500 ms aligned to arrival at the reward spout. For approach we selected an interval of 500 ms extending back in time from touch onset. The fifth episode used for comparison was “baseline,” taken as the interval from 2.5 to 3 s before first contact. The turn episode was not included in the spectral analysis.

### Whisking and LFP Power Spectrograms

Power spectra in the time and frequency domains (4–20 Hz) for whisking and hippocampal LFP were computed using a fast Fourier transform in the Fieldtrip toolbox [67]. Spectrograms were examined for the entire period extending 3 s on either side of first whisker contact, yielding a period that covered pre-trial baseline, approach, stimulus palpation, turn, and reward consumption. To inspect the time course of frequency changes, we computed the power spectrogram using an adaptive sliding time window eight cycles long (Δt = 8/f) multiplied by Slepian tapers [68]. Power was averaged over all trials of all recording sessions and normalized relative to the baseline (taken 2.5 to 3 s before first contact). Each animal’s log-transformed power value (10 * *log*_10_(*LEPpower*)) from all conditions was divided by 4 Hz baseline LFP power (10 * *log*_10_(4*HZ*)), and then the mean and standard deviation of this normalized power were computed across animals for all conditions.

For statistical analysis of LFP power change across episodes, we computed the power spectrum in the frequency domain in each behavioral epoch (approach, touch, and reward) with equal length of 500 ms. Two other episodes were included for comparison; (i) resting and (ii) walking, both in the open arena. We applied a Fourier transform to segments previously multiplied by a Hanning taper. The null hypothesis was that the power spectrum in the frequency domain was the same in each behavioral episode. To robustly estimate the sampling distribution of the test statistic, mean power value, we needed many samples. If the null hypothesis were true, then randomly changing the designation of the episodes would have no effect on the outcome. By randomly shuffling all trials we could generate an arbitrarily large number of datasets. After each of 1,000 shuffles, we calculated the desired test statistics in order to build a probability distribution for the null hypothesis. The ranking of the real test statistic among the shuffled test statistics gave a *p*-value, namely, the proportion of random partitions with mean power value exceeding the observed one. We applied Bonferroni correction for multiple comparisons of different frequencies. We calculated the mean and standard deviation of the peak frequency (maximum value in the theta range) across all rats and computed a one-way ANOVA with multiple comparison tests of all episodes during the discrimination task. The same analysis was done for whisking data.

### Theta-Whisking Synchronization

We measured phase synchronization between oscillations by the Phase Synchronization Index (PSI) [69]) during approach and touch. We first bandpass filtered LFP (*x*) and whisking (*y*) signals in the range 5 to 12 Hz. We use notation of theta for filtered LFP. By applying the Hilbert transform [70,71]) to the filtered data, we extracted instantaneous phase ${(\phi }_{x}^{H},\;\phi_{y}^{H}$). Asymmetries in wave shape of theta oscillation can vary in time and depend on filter settings, instantaneous theta power and frequency. To account for these asymmetries, a conservative approach was taken by testing the distribution of phases in each session for uniformity prior to unit analysis. This distribution then was corrected for the bias with a Ψ-transform [6]. We computed the phase difference between the signals, $\Phi_{xy}^{H}(t)-m\equiv n\Phi_{x}^{H}(t)\Phi_{y}^{H}(t)$. *x* and *y* will be synchronized if the phase difference of their analytic signals remains bounded for all *t*. Because theta and whisking have prominent spectral peaks at a similar frequency, we focused only on the 1:1 (*n*:*m*) locking condition Phase Synchronization Index (PSI) is defined as:

$$PSI=\sqrt{{\langle \text{cos}\theta_{xy}\left(t\right)\rangle }^{2}+{\langle \text{sin}\theta_{xy}\left(t\right)\rangle }^{2}}$$

where the bracket denotes average over time. The PSI varies from 0 (the two signals are not synchronized) to 1 (constant phase difference).

For the two behavioral episodes in which whisking video records were acquired (approach and touch), PSI was computed on every trial, for equal lengths of approach and touch episodes (250 ms approximately) across trials. We generated the confidence limit by a bootstrap method; we computed the PSI of 2,000 sets of theta and whisking data after shuffling signals across trials and, from this, calculated the 95% confidence interval about the population mean (mean PSI_shuffle_ = 0.507). By similar tests, we measured PSI during two episodes—resting and walking—when the rat foraged along an open floor. We tested the consistency of phase delay across trials by the Rayleigh test.

### Spike Phase-Locking Analysis

Once the Hilbert transform assigned instantaneous phase to whisking and to hippocampal theta, barrel cortex units’ spikes could also be assigned both a whisking and theta phase. The principal model against which our data was tested was the von Mises distribution, a unimodal distribution with probability density function *f*(Φ|*θ*, *k*)) = *e* ^*kcos*(Φ−*θ*)^/(2*πI*_*o*_(*k*)), and parameters κ (concentration), θ (mean angle) and I_o_ (modified Bessel function). The function has maximum value at Φ = *θ*. Parameters θ and κ are analogous to the mean and variance in the linear normal distribution. For κ = 0 the von Mises distribution takes the form of a uniform distribution; f(Φ) = 12π. The larger the κ the more the distribution is concentrated around the mean angle, θ. To test data against the uniform distribution we applied the Rayleigh test, using statistic Z defined as *Z* = 2*nr* ^2^, where

$$r\;=\;\sum_{j=1}^{n}\text{cos}\left(\theta_{j}\right)²+\sum_{j=1}^{n}\text{sin}{\left(\theta_{j}\right)}^{2}$$

Given the sum of *n* vectors representing the preferred directions, Z tests whether the mean length *r* is sufficiently large to indicate a non-uniform distribution. The large-sample asymptotic distribution of Z under uniformity is χ^2^ with two degrees of freedom [72]). Uniformity is rejected when Z is far from 0. The alternative hypothesis is that the population has a von Mises distribution with small κ. We note that as mean vector length *r* depends on the sample size, so do all measures derived from *r*. Indeed, the statistical distribution under the null hypothesis is only true asymptotically. For small datasets, the null distribution of Z is typically broad, and therefore the null hypothesis is harder to reject; whereas with a large dataset the distribution can narrow. By varying sample size from 100 to 10,000, values of κ were found to vary from 0.1 to 1.5. We did a simulation [51] to see how the value of κ converges to the asymptote with increasing sample size and settled on the following criteria: neurons emitting fewer than 1,000 spikes per condition were not included in the analysis unless the Rayleigh test was significant. Because sample size has a large effect on the parameters computed, we compared phase modulation between conditions by taking samples of equal size. To avoid any sampling bias, we applied a bootstrap procedure by randomly sampling the original spike pool of each neuron for 500 iterations. In each iteration we calculated the statistics Z and the concentration parameter, *k*; then we calculated the median values using CircStat toolbox [73].

## Supporting Information

S1 Fig Phase-coherence between barrel cortex, whisking cycle, and theta. **(A)** Angle histogram of the phase distribution of all spikes fired by the entire set of neurons (including those not significantly phase locked) in relation to the whisking cycle during approach and touch. Spikes were significantly phase locked to whisking during approach (Rayleigh test, *p* < 0.00001) and touch (Rayleigh test, *p* < 0.00001). The radial axis indicates the spiking probability for the corresponding direction (bin width: 10 degrees). The value 0.03 marks the axis corresponding to the probability value of 3%. Arrows indicate the mean preferred phase across spikes: approach: 2.06 radians, touch: 3.53 radians. **(B)** Angle histogram of the phase distribution of all spikes fired by the entire set of neurons (including those not significantly phase locked) in relation to theta during no task (waiting period), approach and touch. Only approach and touch yielded significant clustering of spikes (Rayleigh test, *p* = 0.62 during pre-trial waiting, *p* < 0.00001 during approach, *p* < 0.000001 during touch). Arrows indicate the mean preferred phase across spikes: approach: 3.22 radians, touch: 3.05 radians. Arrow is not shown in the left plot (waiting for bridge) because preferred theta phase of spiking was not significant. All data are available at http://figshare.com/s/99b31b8a567f11e5b81d06ec4bbcf141.
(TIFF)

S2 Fig Phase-coherence between phase locked barrel cortex neurons, whisking cycle, and theta. **(A)** Mean phase angle histogram for the set of whisking phase-coherent neurons (Rayleigh test, *p* < 0.05) during approach and touch. The radial axis indicates the fraction of neurons with preferred phase of firing for the corresponding direction (bin width: 18 degrees). The arrow in each plot represents the mean preferred phase across all neurons and arrow length is proportional to the concentration parameter κ from the von Mises distribution; the value 0.2 marks the limit corresponding to the fraction of 20% of neurons. **(B)** Mean phase angle histogram for the set of theta phase-coherent neurons (Rayleigh test, *p* < 0.05) during waiting, approach and touch. The conventions follow from those in (A). Arrow not shown for the plot corresponding to waiting period because there was no significant phase clustering for the full set of neurons. All data are available at http://figshare.com/s/99b31b8a567f11e5b81d06ec4bbcf141.
(TIFF)

## Abbreviations

EEG: electroencephalogram
EMG: electromyiography
LFP: local field potential
PPT: peduculopontine tegmental nucleus
PSI: Phase Synchronization Index
RPO: reticularis pontis oralis
vIRt: ventral intermediate reticular formation
