# Supplementary figures and images for: Coherence between Rat Sensorimotor System and Hippocampus Is Enhanced during Tactile Discrimination
Source: PLoS Biol. 2016 Feb 18;14(2):e1002384. doi: 10.1371/journal.pbio.1002384 (PMC4758608; doi:10.1371/journal.pbio.1002384)

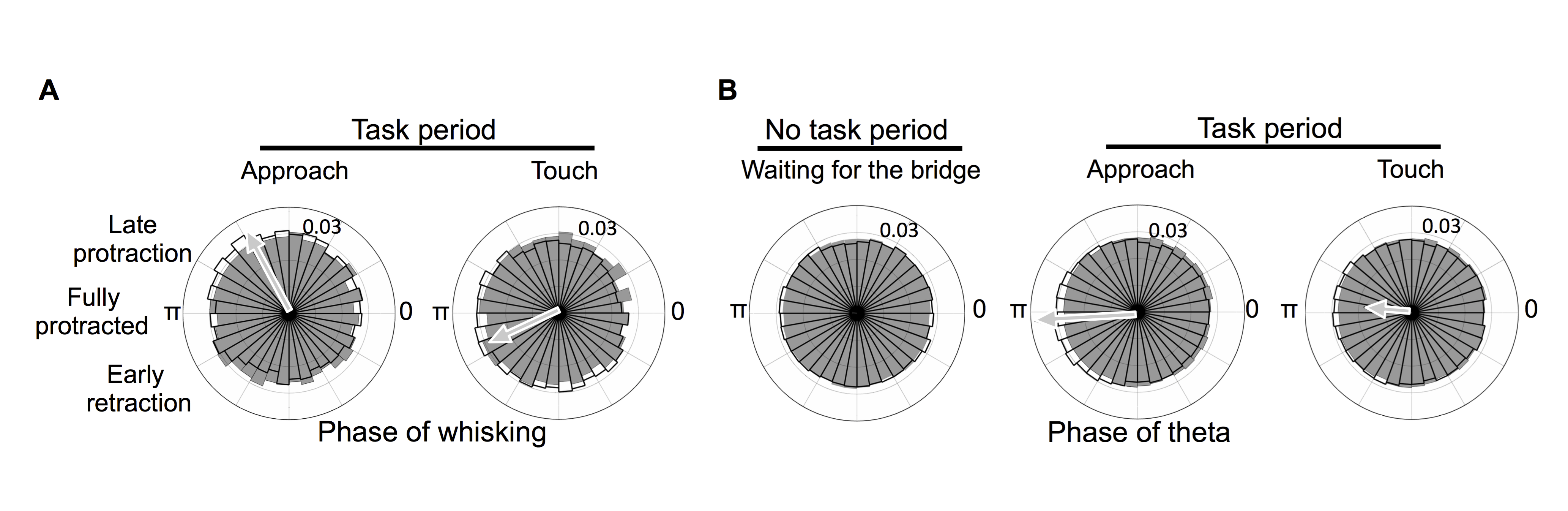

Supplement: S1 Fig — (A) Angle histogram of the phase distribution of all spikes fired by the entire set of neurons (including those not significantly phase locked) in relation to the whisking cycle during approach and touch. Spikes were significantly phase locked to whisking during approach (Rayleigh test, p < 0.00001) and touch (Rayleigh test, p < 0.00001). The radial axis indicates the spiking probability for the corresponding direction (bin width: 10 degrees). The value 0.03 marks the axis corresponding to the probability value of 3%. Arrows indicate the mean preferred phase across spikes: approach: 2.06 radians, touch: 3.53 radians. (B) Angle histogram of the phase distribution of all spikes fired by the entire set of neurons (including those not significantly phase locked) in relation to theta during no task (waiting period), approach and touch. Only approach and touch yielded significant clustering of spikes (Rayleigh test, p = 0.62 during pre-trial waiting, p < 0.00001 during approach, p < 0.000001 during touch). Arrows indicate the mean preferred phase across spikes: approach: 3.22 radians, touch: 3.05 radians. Arrow is not shown in the left plot (waiting for bridge) because preferred theta phase of spiking was not significant. All data are available at http://figshare.com/s/99b31b8a567f11e5b81d06ec4bbcf141. (TIFF) [file pbio.1002384.s001.tiff]

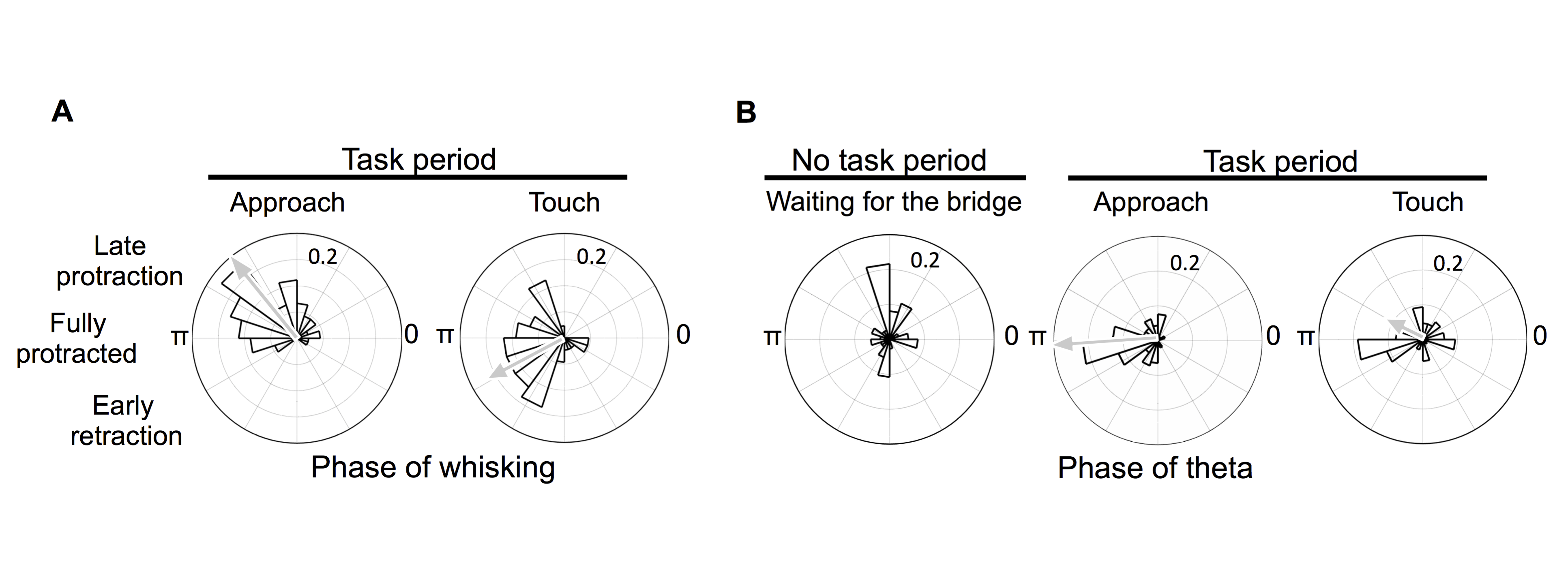

Supplement: S2 Fig — (A) Mean phase angle histogram for the set of whisking phase-coherent neurons (Rayleigh test, p < 0.05) during approach and touch. The radial axis indicates the fraction of neurons with preferred phase of firing for the corresponding direction (bin width: 18 degrees). The arrow in each plot represents the mean preferred phase across all neurons and arrow length is proportional to the concentration parameter κ from the von Mises distribution; the value 0.2 marks the limit corresponding to the fraction of 20% of neurons. (B) Mean phase angle histogram for the set of theta phase-coherent neurons (Rayleigh test, p < 0.05) during waiting, approach and touch. The conventions follow from those in (A). Arrow not shown for the plot corresponding to waiting period because there was no significant phase clustering for the full set of neurons. All data are available at http://figshare.com/s/99b31b8a567f11e5b81d06ec4bbcf141. (TIFF) [file pbio.1002384.s002.tiff]
